# Synergistic neuroprotective effect of rasagiline and idebenone against retinal ischemia-reperfusion injury via the Lin28-let-7-Dicer pathway

**DOI:** 10.18632/oncotarget.24343

**Published:** 2018-01-30

**Authors:** Dawei Lei, Zhengbo Shao, Xinrong Zhou, Huiping Yuan

**Affiliations:** ^1^ Department of Ophthalmology, Second Affiliated Hospital of Harbin Medical University, Harbin 150086, China

**Keywords:** rasagiline, idebenone, Lin28-let-7-Dicer, retinal ischemia-reperfusion

## Abstract

Retinal ischemia-reperfusion (RIR) injury causes neuronal degeneration and initiates various optic nerve diseases. This study aimed to investigate the synergistic neuroprotective effect of rasagiline and idebenone against RIR injury. A combination of rasagiline and idebenone was administered intraperitoneally immediately after establishment of the RIR model. Treatment with the combination of the two drugs resulted in a significant restoration of retinal thickness and retinal ganglion cells. Apoptosis of cells in ganglion cell layers was also ameliorated, suggesting that the effect of the two drugs was synergistic and the expression of brain-derived neurotrophic factor increased. Furthermore, idebenone and rasagiline induced the expression of Lin28A and Lin28B, respectively, which resulted in a reduced expression of microRNAs in the let-7 family and an increased protein output of Dicer. The data obtained from gene overexpression and knockdown experiments indicated that let-7 and Dicer were necessary for the synergistic neuroprotective effect of the two drugs. Our findings suggested that combination therapy with rasagiline and idebenone produced a synergistic effect that ameliorated RIR injury and restored visual function. In addition, the combined treatment provided neuroprotection via enhancement of the selective regulation of let-7 by Lin28A/B. These findings implied that a treatment with the combination of rasagiline and idebenone is a feasible treatment option for optic nerve diseases.

## INTRODUCTION

Retinal ischemia is a consequence of reduced retinal blood circulation, which affects the normal functions of the eye tissues and occurs because of an insufficient supply of nutrients and oxygen to the retina. This results in neuronal damage, morphological degeneration of the retina, and impaired retinal function [1]. Immediate reperfusion rapidly restores the blood supply; however, it causes further damage to the retina by stimulating inflammation and an excessive generation of reactive oxygen species (ROS). This accelerates neuronal cell death [2]. Retinal ischemia-reperfusion (RIR) injury is the final pathological process in ocular diseases such as retinopathy of prematurity, diabetic retinopathy, acute glaucoma, and retinal vascular occlusion [3–6]. RIR injury is also involved in the pathological changes that occur in cerebral vascular diseases and acute ischemic stroke [7, 8]. Thrombolytic treatment of retinal vascular occlusion and ophthalmic operation may disturb intraocular pressure, adversely influence retinal blood flow, and initiate RIR injury. Together with neuronal death and reduced retinal function [9, 10], RIR injury often results in visual impairment and ultimately leads to vision loss owing to a lack of effective treatments.

Several medications such as calcium antagonists, antioxidants, and neurotrophic factors, have been investigated in experimental and clinical research; however, results from these clinical studies have been disappointing, suggesting that it is hard to effectively treat RIR injury [1]. Recent investigations into characterizing mRNA and microRNA expression profiles during RIR injury suggested that impairments in functional interactions involving multiple cellular factors, rather than individual cellular factors, cause phenotypic alterations following RIR injury [11–14]. Therefore, the interactions between cellular factors, instead of the factors themselves, may be more appropriate drug targets. In brain-derived neurotrophic factor (BDNF)-dependent and nondependent interactions, Lin28 mediates let-7 expression and increases the protein output of Dicer. These events cause an increase in mature microRNA levels in neurons and result in cellular repair [15–17]. The work by Tao and his colleagues suggested that the Lin28-let-7-Dicer pathway plays a critical role in retinal neurogenesis and regeneration [18, 19].

Rasagiline is a neuroprotective agent that was initially developed for Parkinson’s disease [20, 21]. It has been confirmed that rasagiline protects the optic nerve from neurodegeneration through its anti-apoptotic activity [22, 23]. Oxidative damage is involved in apoptosis after RIR injury [24]. Antioxidants such as alpha-lipoic acid (ALA), lutein, and idebenone have been shown to have neuroprotective effects on the optic nerve owing to strong antioxidant activity [25–28]. In the present study, an *in vivo* mouse model of RIR injury was established to investigate whether rasagiline targeted the Lin28-let-7-Dicer pathway and provided better retinal ischemia protection if an antioxidant (ALA, lutein, or idebenone) was jointly administered. The results from this study may provide a novel, effective, and feasible therapeutic strategy for RIR injury.

## RESULTS

### Rasagiline and idebenone synergistically protected visual function against RIR injury

**Figure 1:**
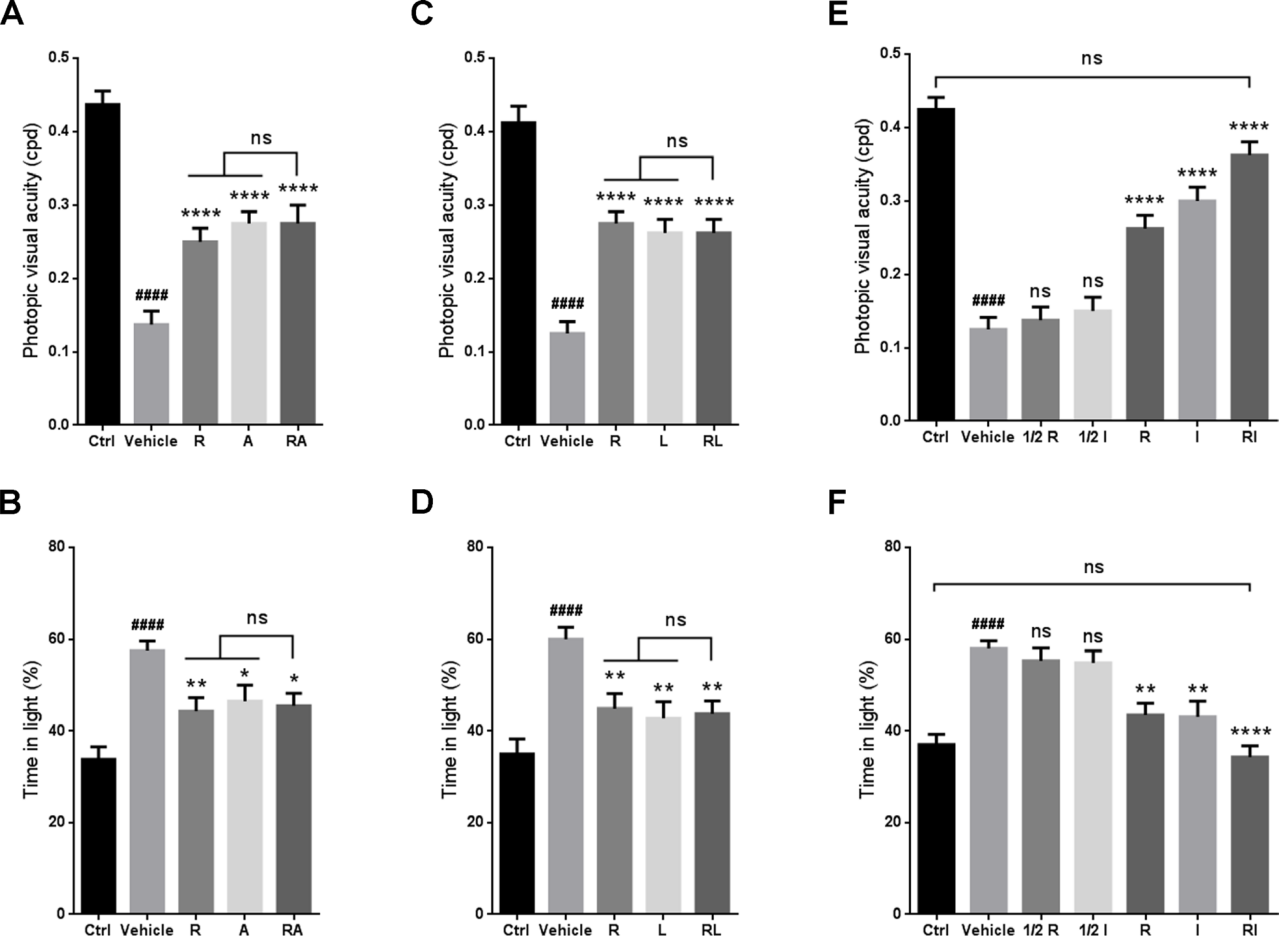
Synergistic effect of rasagiline and idebenone on the protection of visual function against RIR injury (**A**) Photopic visual acuity of RIR-injured mice after treatment with rasagiline, ALA, or both. (**B**) Percent time spent in the light compartment by RIR-injured mice after treatment with rasagiline, ALA, or both. (**C**) Photopic visual acuity of RIR-injured mice after treatment with rasagiline, lutein, or both. (**D**) Percent time spent in the light compartment by RIR-injured mice after treatment with rasagiline, lutein, or both. (**E**) Photopic visual acuity of RIR-injured mice after treatment with rasagiline (0.05 or 0.1 mg·kg^–1^), idebenone (5 or 10 mg·kg^–1^), or both (0.05 mg·kg^–1^ of rasagiline and 5 mg·kg^–1^ of idebenone). (**F**) Percent time spent in the light compartment by RIR-injured mice after treatment with rasagiline (0.05 or 0.1 mg·kg^–1^), idebenone (5 or 10 mg·kg^–1^), or both (0.05 mg·kg^–1^ of rasagiline and 5 mg·kg^–1^ of idebenone). *n* = 8, ^####^*p* < 0.0001, *versus* control; ^*^p < 0.05, ^**^p < 0.01, ^****^p < 0.0001, *versus* vehicle; ns: no significance.

Optomotor response and light-dark exploration tests were performed to evaluate the visual function of the RIR-injured mice when rasagiline was jointly administered with ALA, lutein or idebenone. No significant differences in visual acuity or the percent of time spent in the light chamber were observed between the group treated with rasagiline and ALA and the group treated with rasagiline or ALA (Figure 1A and 1B). Similar results were found for the drug combination of rasagiline and lutein (Figure 1C and 1D). Increased visual acuity was observed in the animals after treatment with a dose of rasagiline or idebenone rather than half a dose of rasagiline or idebenone (Figure 1E). More obvious improvement in visual acuity in mice was provided by joint administration of the two drugs at half doses. The results of light-dark exploration test agreed with this finding. The RIR injured mice treated with rasagiline and idebenone spent less time than that spent by the mice in the other groups (Figure 1F). No significant differences in visual acuity or the percent of time spent in the light chamber were found between the rasagiline and idebenone group and the control group.

### Rasagiline and idebenone synergistically protected the retina against RIR injury

**Figure 2:**
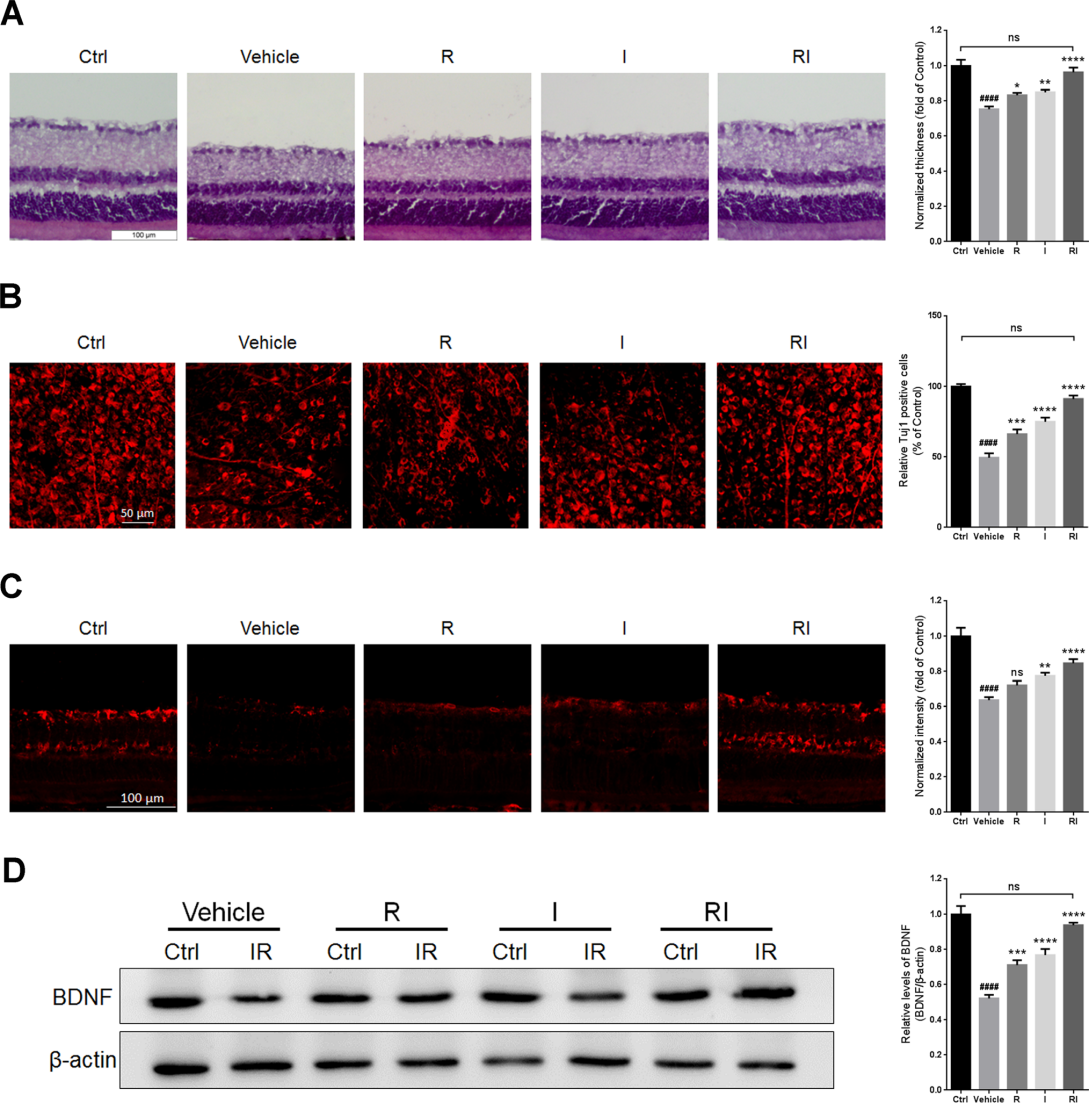
Retinal protection of rasagiline combined with idebenone against RIR injury (**A**) HE staining shows the thickness of retinas at day 7 after IR injury. (**B**) Immunofluorescent staining by Tuj1 on retinal flat mounting displays the survival of RGCs after RIR lesions. (**C**) Immunofluorescent staining displays BDNF levels (red) in the GCLs of the IR-injured retina. (**D**) Western blot analysis for the expression level of BDNF in the whole IR-injured retinas. *n* = 8, ^####^*p* < 0.0001, *versus* control; ^*^p < 0.05, ^**^p < 0.01, ^***^p < 0.001, ^****^p < 0.0001, *versus* vehicle; ns: no significance.

Retinal thickness was evaluated by hematoxylin and eosin (HE) staining. RIR injury led to a decrease in retinal thickness (Figure 2A). The mice treated with rasagiline and idebenone had approximately normal and obviously thicker retinas than did the mice in the other injury groups. RIR injury decreased the density of retinal ganglion cells (RGCs) as determined by Tuj1 staining and retinal flat mounting (Figure 2B). Treatment with rasagiline and idebenone together more effectively increased the number of Tuj1-stained RGCs than administration of rasagiline or idebenone alone. No significant difference was found between the thickness of normal retinas and that of RIR-injured retinas after rasagiline and idebenone were administered. RIR injury decreased BDNF levels in the ganglion cell layers (GCLs) and the whole retinas (Figure 2C and 2D). Treatment with either rasagiline or idebenone increased BDNF levels; however, joint administration of the two drugs led to a reversal of the BDNF expression to normal levels.

### Rasagiline and idebenone in combination reduced oxidative damage and apoptosis

**Figure 3:**
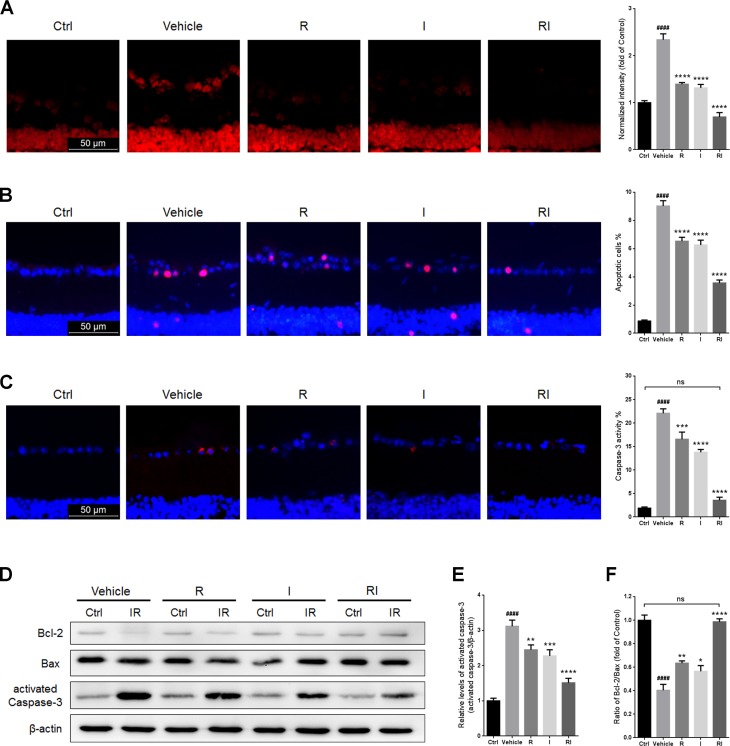
Rasagiline and idebenone as a combination therapy reduce oxidative damage and apoptosis (**A**) DHE staining (red) was performed to detect ROS production in GCLs of IR-injured retinas. (**B**) The percentage of apoptotic cells (pink) was evaluated by TUNEL assay in GCLs of IR-injured retinas. (**C**) The percentage of activated caspase-3-positive cells (red) in GCLs of IR-injured retinas. (**D**) Bcl-2/Bax ratios and activated caspase-3 levels in IR-injured retinas were evaluated by western blot analysis. (**E**) Densitometric analysis of protein expression of activated-caspase-3. (**F**) Densitometric analysis of Bcl-2/Bax ratios. *n* = 8, ^####^*p* < 0.0001, *versus* control; ^*^p < 0.05, ^**^p < 0.01, ^***^p < 0.001, ^****^p < 0.0001, *versus* vehicle; ns: no significance.

RIR injury resulted in a significant increase in ROS levels in GCLs (Figure 3A). ROS levels in GCLs of the injured retinas treated with rasagiline and idebenone in combination were lower than those in retinas treated with rasagiline or idebenone alone (Figure 3A). The percentage of apoptotic cells in the GCLs of the retinas after RIR injury was obviously higher than that in the normal retinas and was decreased after treatment with rasagiline, idebenone, or both (Figure 3B). RIR injury also induced the activation of caspase-3, which is a proteinase that is considered as a marker of apoptosis [29]. The percentage of activated caspase-3-positive cells in the GCLs and the level of activated caspase-3 in the retinas increased after RIR injury, but were obviously reduced after treatment with rasagiline, idebenone, or both (Figure 3C–3E). The anti-apoptotic effect of the combination drug treatment was better than that of monotherapy. The Bcl-2/Bax ratio notably reduced in RIR-injured retinas (Figure 3D and 3F). The ratio in retinas treated with both rasagiline and idebenone was higher than that in single drug-treated retinas and similar to that in normal retinas.

### Rasagiline combined with idebenone upregulated Dicer expression via the Lin28-let-7 pathway

**Figure 4:**
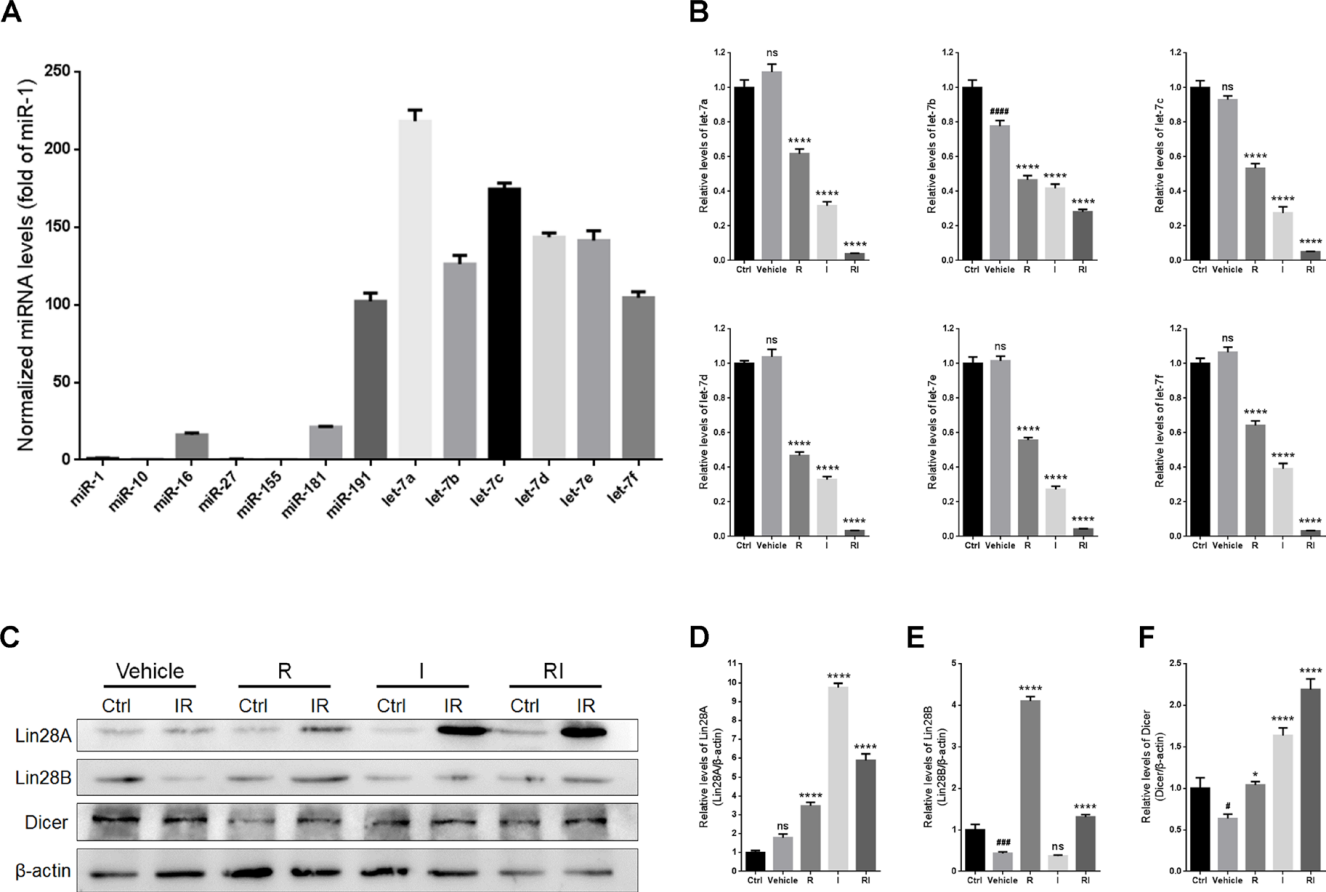
Expressions of Lin28A, Lin28B, Dicer, and the let-7 family in IR-injured retinas (**A**) Levels of microRNAs in the uninjured retinas. (**B**) Levels of let-7 family members in IR-injured retinas. (**C**) Western blot analysis of Lin28A, Lin28B, Dicer, and β-actin from retinas injured for 7 days. (**D**) Densitometric analysis of protein expression of Lin28A in injured retinas. (**E**) Densitometric analysis of Lin28B in injured retinas. (**F**) Densitometric analysis of Dicer in injured retinas. *n* = 8, ^#^*p* < 0.05, ^###^*p* < 0.001, ^####^*p* < 0.0001, *versus* control; ^*^p < 0.05, ^****^p < 0.0001, *versus* vehicle; ns: no significance.

Quantitative real-time polymerase chain reaction (qRT-PCR) was used to assess the expression levels of microRNAs in the retina after RIR injury. The results indicated that the levels of most of the let-7 family members were higher than those of the other microRNAs (Figure 4A). Treatment with either rasagiline or idebenone reduced the expression levels of all the tested let-7 family members after RIR lesions were formed (Figure 4B). Treatment with rasagiline and idebenone together induced an approximately 10-fold decrease in the levels of all the tested let-7 microRNAs, except for let-7b (approximately a 2-fold decrease), in the damaged retinas (Figure 4B).

The expression of the let-7 family of microRNAs is selectively regulated by both Lin28A and Lin28B [30–32]. In the present study, the level of Lin28A in the entire retina was unaltered by RIR injury, but obviously increased after treatment with rasagiline, idebenone, or both (Figure 4C and 4D). Interestingly, the level of Lin28A in the group treated only with idebenone was higher than that in any other treatment group. A notable decrease in the level of Lin28B was observed in the injured retinas (Figure 4C and 4E). The level of Lin28B in the retina increased about 8-fold after treatment with rasagiline alone, whereas treatment with idebenone alone had no effect on the level of Lin28B after RIR injury. In combination, rasagiline and idebenone increased the level of Lin28B after RIR injury.

Dicer is an enzyme encoded by the *DICER1* gene. It cleaves pre-microRNA into mature microRNA, which has been confirmed to be important in retinal development and optic neuroprotection [33, 34]. Let-7 has been reported to repress the translation of mRNA into Dicer at the posttranscriptional level [17]. RIR injury caused a decrease in the level of Dicer in the untreated retinas (Figure 4C and 4F). Treatment with rasagiline, idebenone, or both induced a remarkable increase in the level of Dicer, and the highest level was observed in retinas treated with both drugs (Figure 4C and 4F).

### Let-7d overexpression inhibited the optic neuroprotective effects of rasagiline combined with idebenone via the downregulation of Dicer

**Figure 5:**
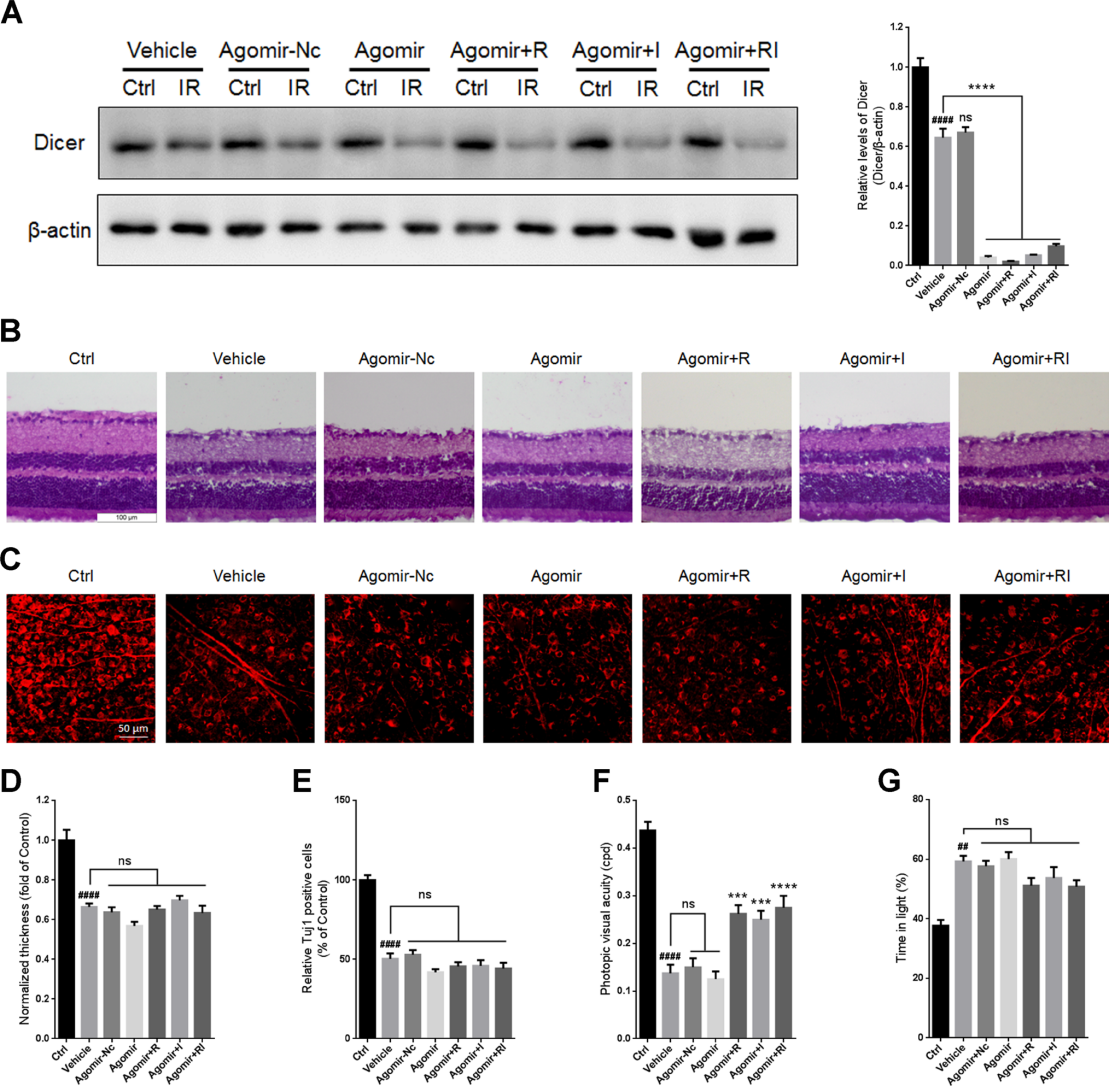
Overexpression of let-7d by agomir in mouse retina (**A**) Western blot for Dicer and β-actin in injured retinas administered with agomir-let-7d or agomir-Nc. (**B**) HE staining shows the thickness of retinas injured for 7 days after let-7d overexpression. (**C**) Immunofluorescent staining by Tuj1 on retinal flat mounting displays the survival of RGCs in IR-injured retinas after let-7d overexpression. (**D**) Analysis of retinal thickness of IR-injured retinas after let-7d overexpression. (**E**) Analysis of the survival of RGCs in IR-injured retinas by Tuj1 immunostaining on retinal flat mounting after let-7d overexpression. (**F**) Photopic visual acuity of RIR-injured mice after let-7 overexpression. Data were shown as mean ± SEM (**G**) Percent time spent in the light chamber by RIR-injured mice after intravitreal injection of let-7d Agomir. *n* = 8, ^##^*p* < 0.01, ^####^*p* < 0.0001, *versus* control; ^***^p < 0.001, ^****^p < 0.0001, *versus* vehicle; ns: no significance.

To investigate whether the combination of rasagiline and idebenone plays a neuroprotective role via downregulating let-7 microRNAs, an agomir for let-7d was designed and injected into the vitreous chambers of mouse eyes to selectively overexpress let-7d before RIR injury was induced. The results indicated that no significant difference in the level of Dicer was observed between untreated retinas and agomir-Nc-treated retinas at day 7 after RIR injury (Figure 5A). The level of Dicer was further decreased in the agomir-let-7d treated retinas after RIR lesions had formed and did not respond to treatment with rasagiline, idebenone, or both (Figure 5A).

There were no significant differences in retinal thickness or the survival of RGCs between the vehicle group and the agomir-let-Nc-treated group. Treatment with agomir-let-7d did not alter retinal thickness or the number of RGCs. Treatment with rasagiline, idebenone, or both did not increase retinal thickness or the number of RGCs after let-7d overexpression (Figure 5B–5E).

Accordingly, no significant difference in visual acuity was observed between untreated mice and agomir-Nc-treated mice. Agomir-let-7d did not alter the visual acuity of mice after RIR injury. The visual acuity in agomir-let-7d-treated mice treated with rasagiline, idebenone, or both partly increased compared with that in control mice (Figure 5F). Light-dark exploration testing indicated that there was no significant difference in time spent in the light chamber between the vehicle group and any of the agomir-treated groups (Figure 5G). Treatment with rasagiline, idebenone, or both did not influence the time spent in the light by the mice after let-7d overexpression.

**Figure 6:**
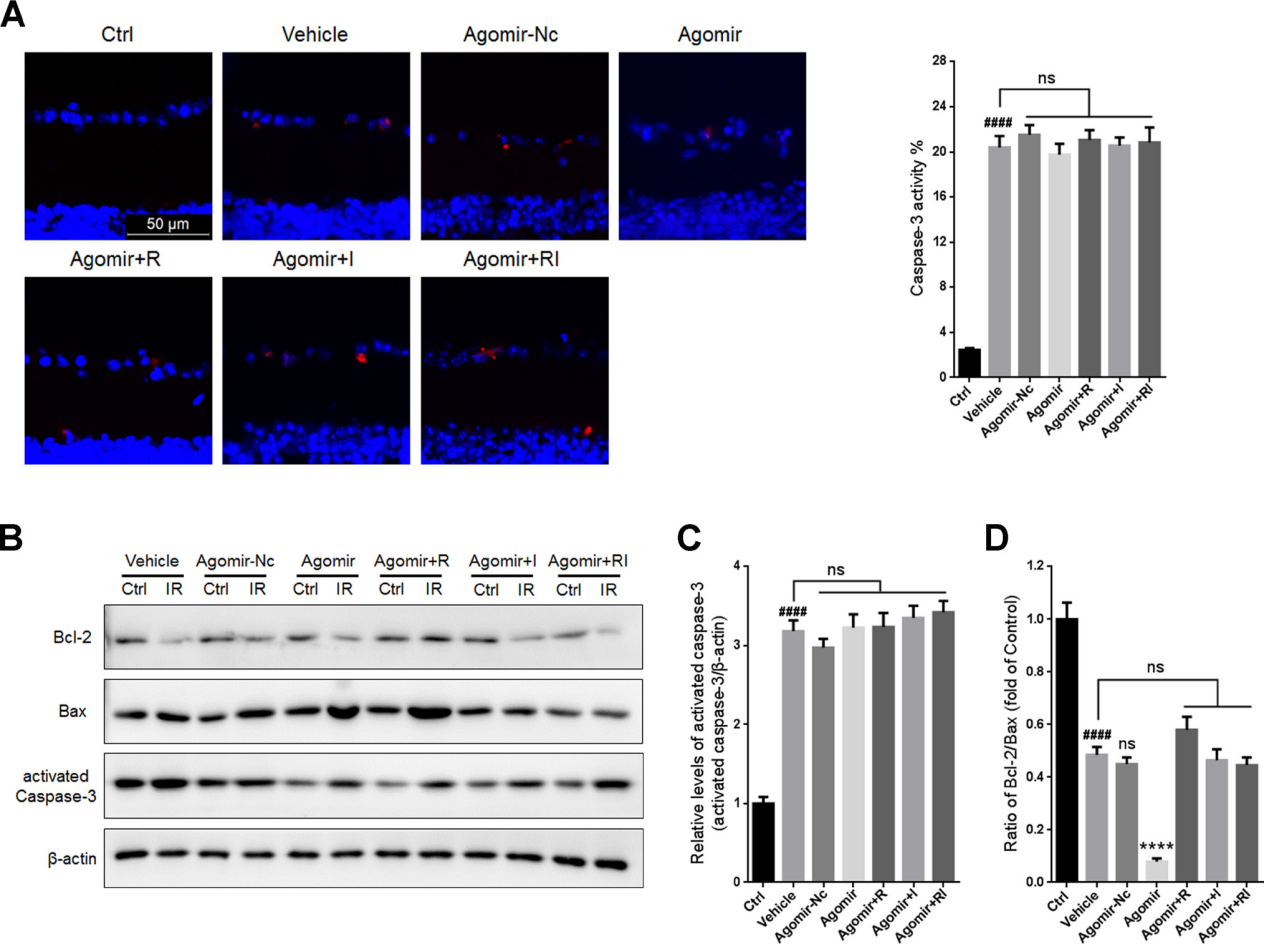
Apoptosis in IR-injured retinas after overexpression of let-7d by agomir (**A**) The percentage of activated caspase-3-positive cells (red) in GCLs of IR-injured retinas after let-7d overexpression. (**B**) Bcl-2/Bax ratios and activated caspase-3 in IR-injured retinas were evaluated by western blot analysis after let-7d overexpression. (**C**) Densitometric analysis of expression of activated caspase-3 in IR-injured retinas after let-7d overexpression. (**D**) Densitometric analysis of Bcl-2/Bax ratios in IR-injured retinas after let-7d overexpression. *n* = 8, ^####^*p* < 0.0001, *versus* control; ^****^p < 0.0001, *versus* vehicle; ns: no significance.

Treatment with agomir-Nc or agomir-let-7d did not significantly alter the percentage of activated caspase-3-positive cells in the GCLs or the levels of activated caspase-3 in the ischemia-reperfusion (IR)-damaged retinas. Furthermore, let-7d overexpression did not influence the activity of caspase-3 in the injured retina regardless of whether rasagiline, idebenone, or both were administered (Figure 6A–6C). Treatment with agomir-Nc did not affect the Bcl-2/Bax ratio in IR-injured retinas. Let-7d overexpression induced a further decrease in the Bcl-2/Bax ratio and counteracted the effect of the combination of rasagiline and idebenone by increasing the Bcl-2/Bax ratio in IR-damaged retinas (Figure 6B and 6D).

### Dicer inhibition prevented the optic neuroprotective effects of rasagiline combined with idebenone

**Figure 7:**
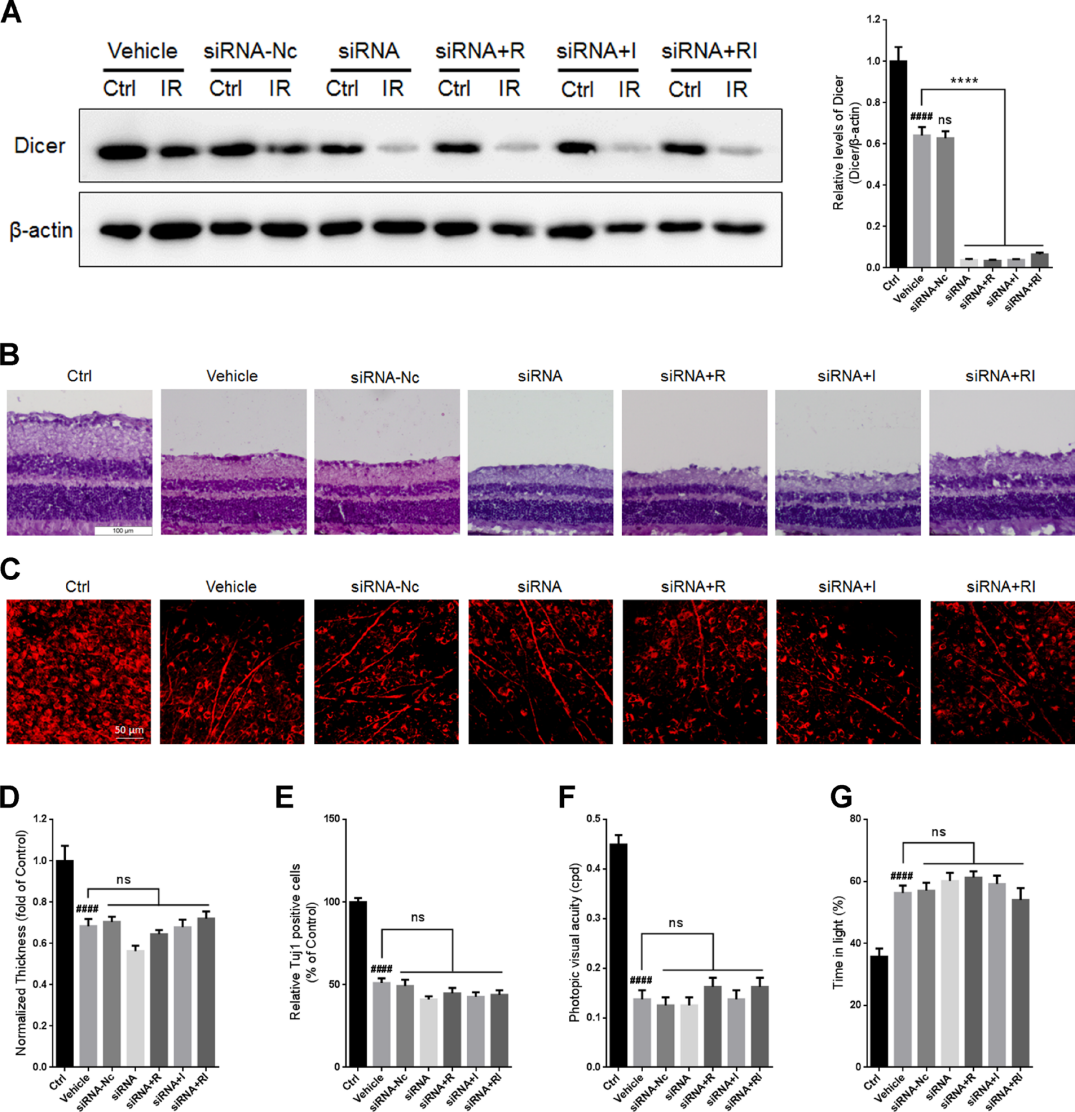
*DICER1* knockdown by siRNA in mouse retina (**A**) Western blot analysis for Dicer and β-actin in injured retinas after *DICER1* knockdown in the eyes. (**B**) HE staining shows the thickness of retinas injured for 7 days after inhibition of Dicer by siRNA. (**C**) Immunofluorescent staining of Tuj1 on retinal flat mounting displays the survival of RGCs in IR-injured retinas after *DICER1* knockdown. (**D**) Analysis of retinal thickness of IR-injured retinas after *DICER1* knockdown. (**E**) Analysis of the survival of RGCs in IR-injured retinas by Tuj1 immunostaining on retinal flat mounting after *DICER1* knockdown. (**F**) Photopic visual acuity of RIR-injured mice after inhibition of Dicer by siRNA. (**G**) Percent time spent in the light chamber by RIR-injured mice after administrated with siRNA for *DICER1*. *n* = 8, ^####^*p* < 0.0001, *versus* control; ^****^p < 0.0001, *versus* vehicle; ns: no significance.

To investigate whether the combination of rasagiline and idebenone plays a neuroprotective role via upregulating Dicer, an siRNA for *DICER1* was designed and injected into the vitreous chambers of mouse eyes to selectively inhibit its expression. There was no significant difference in the level of Dicer between untreated retinas and siRNA-Nc-treated retinas (Figure 7A). Treatment with siRNA for *DICER1* resulted in a further decrease in Dicer levels after RIR lesions were formed (Figure 7A). Treatment with rasagiline, idebenone, or both did not alter the expression of Dicer in IR-injured retinas after inhibition of Dicer. No significant difference in retinal thickness or RGCs survival was observed between untreated retinas and siRNA-Nc-treated retinas after RIR injury (Figure 7B–7E). Treatment with siRNA for *DICER1* did not alter retinal thickness or the survival of RGCs in IR-injured retinas (Figure 7B–7E). Retinal thickness and the survival of RGCs in retinas treated with rasagiline, idebenone, or both were not altered after Dicer knockdown (Figure 7B-–7E). The optomotor response test indicated no significant difference in visual acuity between untreated mice and siRNA-Nc-treated mice (Figure 7F). *DICER1* knockdown did not alter the visual acuity of RIR-injured mice. Treatment with rasagiline, idebenone, or both did not increase the visual acuity of RIR-injured mice administered siRNA for *DICER1*. Similar results were observed for the light-dark exploration testing (Figure 7G).

**Figure 8:**
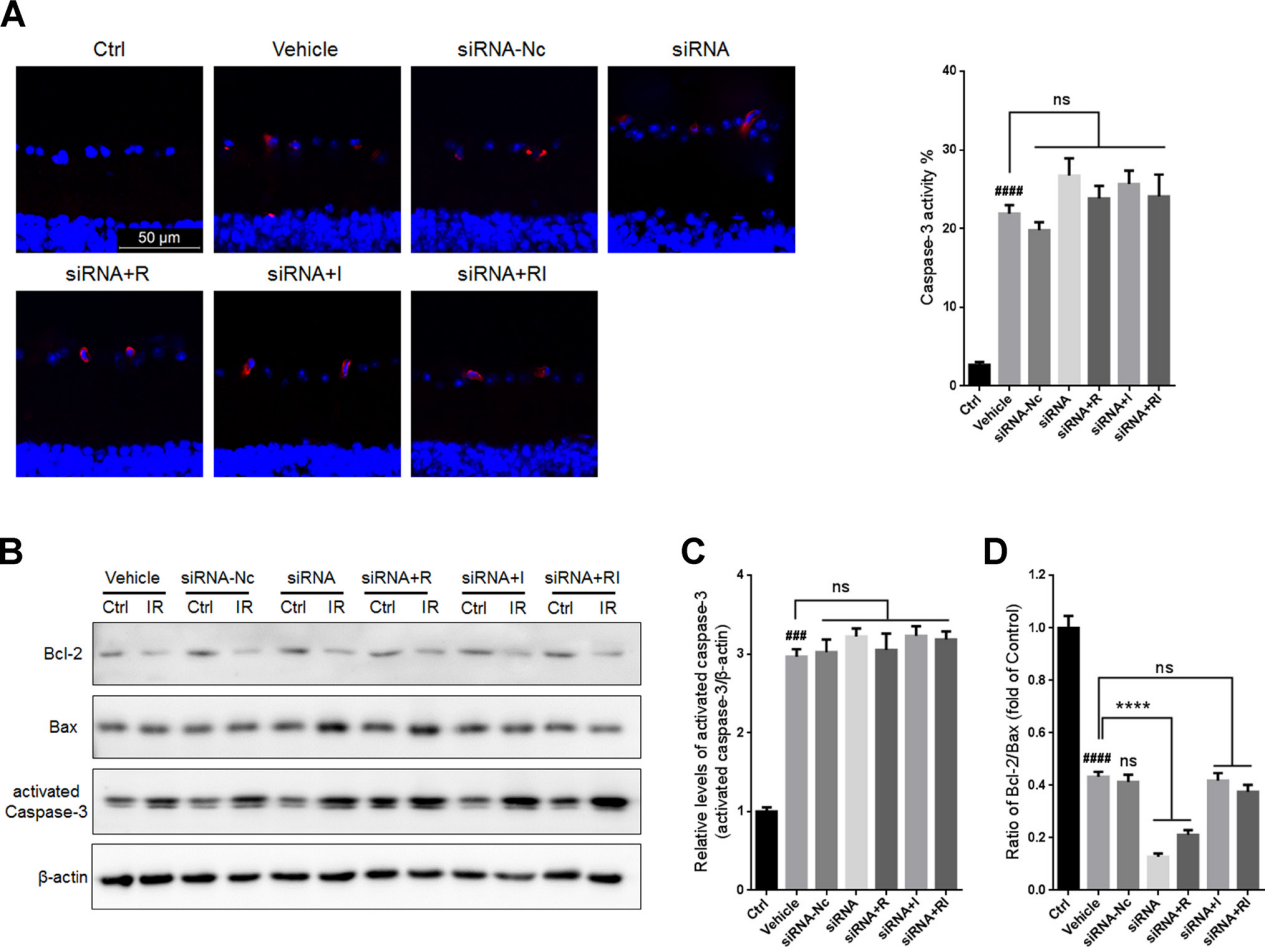
Apoptosis in IR-injured retinas after inhibition of Dicer by siRNA (**A**) The percentage of activated caspase-3-positive cells (red) in GCLs of IR-injured retinas after inhibition of Dicer. (**B**) Western blot analysis for Bcl-2/Bax ratios and activated caspase-3 levelsin IR-injured retinas after administration of siRNA for *DICER1*. (**C**) Densitometric analysis of protein expression of activated caspase-3 in IR-injured retinas after *DICER1* knockdown. (**D**) Densitometric analysis of Bcl-2/Bax ratios in IR-injured retinas after *DICER1* knockdown. *n* = 8, ^###^*p* < 0.001, ^####^*p* < 0.0001, *versus* control; ^****^p < 0.0001, *versus* vehicle; ns: no significance.

No significant difference was found in the percentage of caspase-3 positive cells in GCLs or the level of activated caspase-3 in IR-injured retinas between the vehicle group and the siRNA-Nc-treated group (Figure 8A–8C). Administration of siRNA for *DICER1* did not significantly alter the activity of caspase-3 in IR-injured retinas. In addition, treatment with rasagiline, idebenone, or both did not alter the activity of caspase-3 in IR-damaged retinas after Dicer knockdown. The ratio of Bcl-2/Bax in siRNA-Nc-administered retinas was similar to that in untreated retinas after RIR lesions were formed. *DICER1* knockdown caused a further decrease in the Bcl-2/Bax ratio in IR-injured retinas (Figure 8B and 8D). Treatment with rasagiline, idebenone, or both did not increase the Bcl-2/Bax ratio in IR-injured retinas compared with untreated retinas after *DICER1* knockdown.

### Overdose of rasagiline and idebenone in combination failed to protect the retina against RIR injury

**Figure 9:**
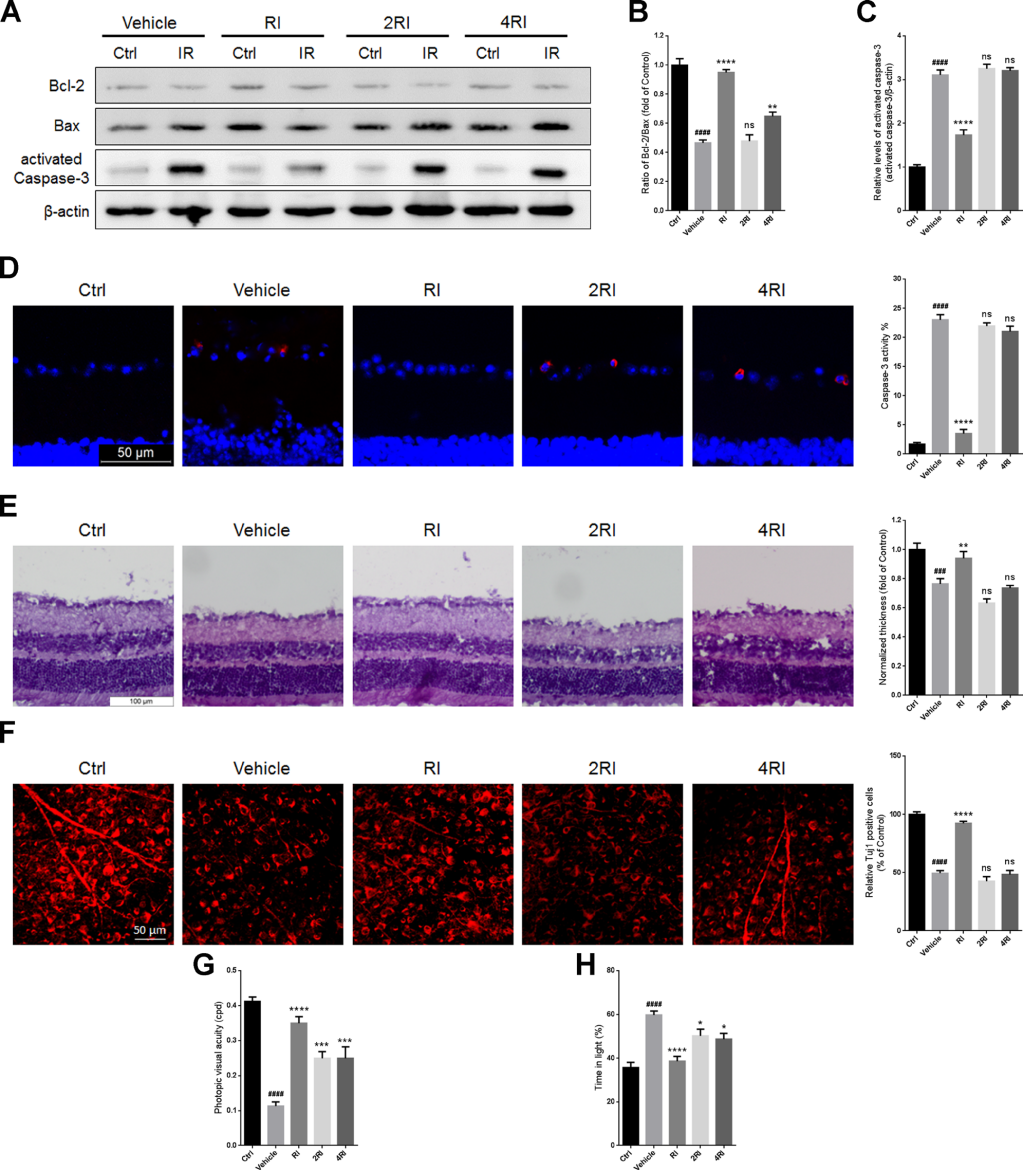
Overdose of rasagiline and idebenone in combination fails to protect the retina against RIR injury (**A**) Western blot analysis for Bcl-2/Bax ratios and activated caspase-3 levels in IR-injured retinas after administration with overdose of rasagiline and idebenone. (**B**) Densitometric analysis of Bcl-2/Bax ratios in IR-injured retinas after administration with overdose of rasagiline and idebenone. (**C**) Densitometric analysis of expression of activated caspase-3 in IR-injured retinas after administration with overdose of rasagiline and idebenone. (**D**) The percentage of activated caspase-3-positive cells (red) in GCLs of IR-injured retinas after treatment with overdose of rasagiline and idebenone in combination. (**E**) Analysis of retinal thickness of IR-injured retinas by HE staining after treatment with overdoses of rasagiline and idebenone. (**F**) Analysis of the survival of RGCs in IR-injured retinas by Tuj1 immunostaining on retinal flat mounting after administration with overdose of rasagiline and idebenone. (**G**) Photopic visual acuity of RIR-injured mice treated with overdosed rasagiline and idebenone. (**H**) Percent time spent in the light chamber by RIR-injured mice after overdosed administrations of rasagiline and idebenone. *n* = 8, ^###^*p* < 0.001, ^####^*p* < 0.0001, *versus* control; ^*^p < 0.05, ^**^p < 0.01, ^***^p < 0.001, ^****^p < 0.0001, *versus* vehicle; ns: no significance.

The Bcl-2/Bax ratios in retinas of mice administered with high doses of rasagiline and idebenone were lower than those of mice treated with low dose combination after RIR injury (Figure 9A and 9B). In addition, treatment with high doses of rasagiline and idebenone did not reduce the percentage of activated caspase-3-positive cells in the GCLs or the activated caspase-3 levels of the IR-damaged retinas (Figure 9A, 9C, and 9D). High dose combinations of rasagiline and idebenone were ineffective for increasing retinal thickness or RGCs survival after RIR injury (Figure 9E and 9F) and partly increased the visual acuity of RIR-damaged mice (Figure 9G). Similar results were also observed for the light-dark exploration testing (Figure 9H).

## DISCUSSION

Network-like interactions among genes and microRNAs may contribute to the complexity of the mechanisms underlying RIR injury. Regulation of the nervous system has been suggested to remain robust even under pathological conditions [35, 36]. To overcome a biological system’s robustness, a drug should be able to act on multiple targets that are associated with each other rather than on a single target [37, 38]. Based on this knowledge, a signaling cascade including Lin28A, Lin28B, the let-7 family of microRNAs, and Dicer was investigated in the present study as an integral drug target in a mouse model of acute RIR injury. Activation of the functional association between Lin28A and Lin28B increases the protein output of Dicer and the level of mature microRNAs in neurons and during nerve repair procedures [15, 17, 39]. In our study, a significant downregulation of the signaling cascade was observed, although not all elements in the cascade were downregulated. Our findings indicated the involvement of this cascade in RIR injury.

Rasagiline, idebenone, lutein, and ALA are all neuroprotective agents [25–28, 40, 41]. Any of these drugs, when used alone, was shown to effectively protect visual functions after RIR injury. However, our results indicated that rasagiline combined with idebenone rather than lutein or ALA synergistically protected the visual functions against RIR injury. Therefore, the combination of rasagiline and idebenone were optimized as the experimental tool for inhibiting the robustness of the signaling cascade. When low doses of rasagiline and idebenone were administered in combination, we observed that their joint effect on the neuroprotection and inhibition of the robustness of the signaling cascade was markedly significant and included a significant restoration of retinal thickness, enhancement of RGCs survival, amelioration of cell apoptosis in GCLs, and improvement in visual function. In addition, we found that a low-dose combination of the drugs was superior to a high-dose combination for ameliorating RIR-induced morphological and functional injury in the retina. These findings suggested that the two drugs acted synergistically.

Combination treatment with rasagiline and idebenone reduced caspase-3 and ROS levels, indicating a protective role of the drug combination on mitochondria whose functions were impaired by RIR injury [42]. We observed that idebenone increased the expression levels of Lin28A, whereas rasagiline markedly increased the protein output of Lin28B. The consequences of the aforementioned effects of the drugs were a substantial decrease in the expression levels of the let-7 family of microRNAs and an activation of Dicer, which is an important target gene of let-7a/c/d [17]. Recently, Wang *et al.* demonstrated that a knockdown in let-7a gene expression resulted in protection against cerebral IR injury [43]. This result is consistent with our findings that substantially lowering let-7 expression relieved the IR-induced retinal damage. Our results suggested that downregulation of the let-7 family of microRNAs contributed to retinal protection by drugs in RIR injury, even though dysregulation of let-7s is not likely to be a trigger of RIR-induced impairments. Let-7 is more highly expressed in the retina than other microRNAs, indicating its importance in regulating retinal function. Because all let-7 family members are highly conserved in both sequences and functions in mammals [18], we selected let-7d for overexpression and investigated the effects of the drug combination on the above-mentioned signaling cascade by overexpressing let-7d in the retina with an agomir injection. The overexpression of let-7d completely offset the effect of the drugs on Dicer, retinal thickness, RGCs survival, and apoptosis. These findings implied that lowering let-7 microRNA levels is essential for the optic nerve-protective effects of rasagiline and idebenone.

Furthermore, an siRNA for *DICER1* was designed for our *in vivo* studies. This siRNA has been reported to be stabilized and suitable for *in vivo* studies [44]. The results indicated that Dicer played an important role in the protective effects of the drug combination against RIR injury because the *DICER1* knockdown completely eliminated the drug effects. Our findings were consistent with those of previous studies, which showed that Dicer was essential for retinal development and that Dicer knockout caused functional and structural degeneration of the retina [33, 45].

In summary, we have demonstrated that a low-dose combination of rasagiline and idebenone may be a beneficial treatment for RIR injury. Combination treatment with rasagiline and idebenone resulted in synergistic neuroprotection against RIR injury. The mechanism underlying the observed effect may have arisen from the joint effect of the two drugs on the activation of a signaling cascade composed of Lin28A/B, let-7, and Dicer. The present study also highlighted the critical roles of the Lin28-let-7-Dicer axis in the pathophysiology of RIR injury.

## MATERIALS AND METHODS

### Animals

Healthy male C57BL/6 mice weighing 20–30 g were purchased from the Laboratory Animal Center of Harbin Medical University (Harbin, China). The animals were treated with approval from the Institutional Animal Care and Use Committee of the Harbin Medical University, in accordance with the Statement for the Use of Animals in Ophthalmic and Vision Research by the Association for Research in Vision and Ophthalmology. The animals were housed under the following controlled conditions: 12-h light/dark cycle (8:00–20:00 h light; 20:00–8:00 h dark), temperature of 23–25° C, and humidity of 55 ± 10%. The animals were also given free access to standard food and drinking water.

### RIR injury model

Anesthesia was induced with an intraperitoneal (i.p.) injection of 5% chloral hydrate. Corneal analgesia was achieved using 1 or 2 drops of 0.4% oxybuprocaine hydrochloride. The anterior chamber of the left eye was cannulated with a 30-gauge needle attached to an infusion line from a bottle of balanced salt intraocular irrigating solution. Retinal ischemia was induced by elevating the intraocular pressure to 110 mmHg for 45 min (by lifting the container). Retinal ischemia was confirmed by the whitening of the iris and the loss of red reflex under a microscope. After 45 min of ischemia, the needle was withdrawn from the anterior chamber and reperfusion of the retinal vasculature was confirmed by examination of the fundus. Body temperature was maintained at approximately 36–37° C using an animal temperature controller and heating pad throughout the experiment and until the animals recovered from the anesthesia. Ofloxacin ophthalmic gel (Dikeluo Ofloxacin Eye Ointment; Sinqi Pharmaceutical Co., Ltd., Shenyang, China) was applied topically to the left eye before and after cannulation of the anterior chamber.

### Intravitreal injection of microRNA agomir and siRNA

The agomir for let-7d (micrON™ mmu-let-7d-5p agomir; RiboBio Co., Ltd., Guangzhou, China) was dissolved in sterilized physiological saline to obtain a concentration of 1 nmol·μL^–1^. A 3-μL aliquot of the agomir-let-7d solution was administered to each animal by a unilateral intravitreal injection 24 h before and 3 days after the ischemic injury was induced. The agomir for let-7d was synthesized based on the mature microRNA sequence of mmu-let-7d-5p (5′-AGAGGUAGUAGGUUGCAUAGUU-3′). The negative control agomir (agomir-Nc), siRNA for *DICER1*, and negative control siRNA (siRNA-Nc) were all dissolved and injected in the same manner. All agomirs and siRNAs were purchased from RiboBio Co., Ltd. (Guangzhou, China).

### Group assignment and drug administration

In the ischemic *in vivo* study, rasagiline (HY-14605; MedChem Express, Monmouth Junction, NJ, USA), idebenone (HY-N0303, MedChem Express), ALA (HY-N0492; MedChem Express), and lutein (Catalog No. 07168; Sigma-Aldrich Co, St. Louis, MO, USA) were dissolved in dimethyl sulfoxide (DMSO), then diluted with physiological saline to obtain the desired concentrations. The final concentration of DMSO was 0.1%. The animals were randomly assigned to one of the groups indicated in Table 1. Drugs or vehicle (DMSO+saline) were injected i.p. in a volume of 10 mL·kg^-1^. The respective treatments were administered immediately after reperfusion, then daily after the RIR injury was induced until the animals were sacrificed. Mice from each group were sacrificed 24 h after RIR injury and their eyeballs were harvested to evaluate apoptosis, caspase-3 activity, Bcl2/Bax, and ROS production. At day 7 after RIR lesion induction, mice were randomly chosen from each group for behavioral assays, histopathological examination, BDNF and Tuj1 immunostaining, qRT-PCR, and western blot for BDNF, Lin28A, Lin28B, and Dicer.

**Table 1:** Group assignment and drug administration for mice

Group	Drug administration
Vehicle	DMSO + saline
R	rasagiline (0.1 mg·kg^–1^)
I	idebenone (10 mg·kg^–1^)
L	lutein (0.2 mg·kg^–1^)
A	alpha-lipoic acid (100 mg·kg^–1^)
RI	rasagiline (0.05 mg·kg^–1^) + idebenone (5 mg·kg^–1^)
RL	rasagiline (0.05 mg·kg^–1^) + lutein (0.1 mg·kg^–1^)
RA	rasagiline (0.05 mg·kg^–1^) + alpha-lipoic acid (50 mg·kg^–1^)
1/2R	rasagiline (0.05 mg·kg^–1^)
1/2I	idebenone (5 mg·kg^–1^)
2RI	rasagiline (0.1 mg·kg^–1^) + idebenone (10 mg·kg^–1^)
4RI	rasagiline (0.2 mg·kg^–1^) + idebenone (20 mg·kg^–1^)
Agomir	DMSO + saline + agomir-let-7d
Agomir-Nc	DMSO + saline + agomir-Nc
Agomir+R	rasagiline (0.1 mg·kg^–1^) + agomir-let-7d
Agomir+I	idebenone (10 mg·kg^–1^) + agomir-let-7d
Agomir+RI	rasagiline (0.05 mg·kg^–1^) + idebenone (5 mg·kg^–1^) + agomir-let-7d
siRNA	DMSO + saline + siRNA for *DICER1*
SiRNA-Nc	DMSO + saline + siRNA-Nc
siRNA+R	rasagiline (0.1 mg·kg^–1^) + siRNA for *DICER1*
siRNA+I	idebenone (10 mg·kg^–1^) + siRNA for *DICER1*
siRNA+RI	rasagiline (0.05 mg·kg^–1^) + idebenone (5 mg·kg^–1^) + siRNA for *DICER1*

### Histopathological examination

At day 7 after RIR injury, each eyeball was enucleated and fixed in 4% paraformaldehyde at 4° C overnight. After fixation, the anterior segment of the eyeball was removed and the posterior segment was dehydrated in a graded sucrose series and embedded in O.C.T. compound (Catalog No. 4583; Sakura Finetek Co., Ltd., Tokyo, Japan). For HE staining, sections through the optic disc of the eye were cut into 6-µm-thick sections. Because retinal thickness in the mouse model is not uniform and is dependent upon location, all measurements were performed in the mid-peripheral retinal region 1 mm from the optic nerve head. All images were captured using a charge-coupled device camera (Olympus DP25; Olympus Corporation, Tokyo, Japan) connected to a light microscope (Olympus BX41; Olympus Corporation). The images were analyzed using Image-Pro Plus software (version 6.0.0.260; Media Cybernetics Inc., Bethesda, MD, USA). To assess ischemic damage in the retina, we measured the retinal thickness from the GCL to the outer plexiform layer. Retinal thickness was calculated as the average of six measurements in each eye. Eight mice from each group were histopathologically examined.

### qRT-PCR analysis

Total RNA was isolated using an Ultrapure RNA Kit (CW0581M; CoWin Biotech Co., Beijing, China) according to the manufacturer’s instructions. Reverse transcription to generate cDNA and qRT-PCR was performed using an All-in-One microRNA qRT-PCR Detection Kit (AOMD-Q060; GeneCopoeia Inc., Guangzhou, China). The fold differences between the levels of various transcripts were calculated with the CT method using Mouse snRNA U6 (MmiRQP9002; GeneCopoeia Inc.) as the internal control. After PCR, a melting curve was constructed in the range of 60–95° C to assess the specificity of the amplification products. All the primers in the study were purchased from GeneCopoeia Inc. Eight mice from each group were collected for qRT-PCR analysis.

### ROS formation

Superoxide production was evaluated in fresh frozen retina sections (6-μm thick) using the DHE method (D11347; Molecular Probes, Eugene, OR, USA). Frozen sections were preincubated for 20 min with 10 μM MnTmPyP (Sapphire Bioscience, Waterloo, Australia), which is a superoxide dismutase mimetic, and used as the background controls. The sections were reacted with DHE (5 μM) at 37° C for 30 min in a humidified chamber protected from light. DHE was oxidized on reaction with the superoxide to ethidium bromide, which complexed with DNA in the nucleus and was excited at 488 nm with an emission spectrum at 610 nm [46]. Images of the retina sections were viewed under a fluorescence microscope (Leica DM4000B; Leica Microsystems GmbH, Wetzlar, Germany). All the images were taken using uniform exposure settings (100 ms). The fluorescence intensities of the GCLs in retina sections were measured using Image-Pro Plus software. Eight mice from each group were collected for DHE staining.

### TUNEL assay

Apoptosis in the frozen retina sections (6-μm thick) collected at 24 h after RIR injury was assessed using an *In Situ* Cell Death Detection Kit, POD (Catalog No. 11684817910; Roche Diagnostics Ltd., Shanghai, China) according to the manufacturer’s instructions. The percentage of apoptotic cells in the RGC layer of each specimen was quantified (from the counts of total and TUNEL-positive cells) from 20 images. Eight mice from each group were collected for the TUNEL assay.

### Immunohistochemistry

Eyeballs and retinal cryostat sections (6-μm thick) were collected as described above. Retina sections from each sample were permeabilized with 0.1% Triton X-100 at 25° C for 30 min and blocked in 10% normal donkey serum at 25° C for 60 min. The sections were then incubated in a humidified box at 4° C overnight in anti-BDNF antibody (1:100; ab108319; Abcam, Cambridge, MA, USA) and anti-active caspase-3 antibody (1:100, ab2302, Abcam). The next day, sections were washed with phosphate-buffered saline (PBS) and incubated with Alexa Fluor 594-conjugated secondary antibody (1:200; SA00006-8; Proteintech Group Inc., Chicago, IL, USA) at 25° C for 60 min. Thereafter, the sections were stained with DAPI (C1005; Beyotime Co., Shanghai, China) at 25° C for 5 min and placed in a mounting medium. Images of the sections were obtained using the Leica fluorescence microscope. All the images were taken using uniform exposure settings. The fluorescence intensity and cell count in each section were determined using Image-Pro Plus software. Eight mice from each group were collected for immunohistochemistry analysis.

### Retinal flatmounting, Tuj1 labeling, and counting

To evaluate the density of RGCs, retinal flat mounting and Tuj1 labeling were performed according to a previously published method [47–49]. The eyeballs were placed in 4 % paraformaldehyde (PFA) for 30 min and the whole retina was then carefully dissected and flattened. Thereafter, retinas were blocked in PBS containing 0.1% Triton X-100 and 5% bovine serum albumin (BSA) at 4° C overnight. Subsequently, the retinas were incubated with anti-Tuj1 (1:100, RGCs marker, 10094-1-AP, Proteintech Group Inc.) for 48 h at 4° C. After three washing steps, the retinas were incubated with Alexa Fluor 594-conjugated secondary antibody (1:100; SA00006-8; Proteintech Group Inc.) for 2 h at 25° C and subsequently washed with PBS. The retinas were then mounted on microscope slides. To evaluate the density of RGCs, each retinal quadrant was divided into eight distinct areas of 0.09 mm^2^. The regions of each retina were examined by confocal microscope (Olympus FV1000; Olympus Corporation). The mean density of RGCs per retina was determined by the averages of these areas. Eight mice from each group were collected for retinal flatmounting and Tuj1 labeling.

### Protein extraction and western blot

Retinas were lysed in RIPA lysis buffer (P0013B, Beyotime Co.) and protein extraction was performed according to the manufacturer’s instructions. Protein concentrations were determined using a BCA Protein Assay Kit (P0010, Beyotime Co.). The protein samples were subjected to 8% and 12% sodium dodecyl sulfate polyacrylamide gel electrophoresis using Mini-Protean Tetra Cell (Catalog No. 165-8034; Bio-Rad Laboratories Co., Ltd., Shanghai, China) according to distinct molecular weights. The samples were then electrotransferred to polyvinylidene fluoride (PVDF) membranes using Mini Trans-Blot cells (Catalog No. 170-3930, Bio-Rad Laboratories Co., Ltd.). The membranes were blocked with 5% skimmed milk at 37° C for 1 h, then incubated at 4° C overnight with rabbit anti-BDNF antibody (1:1000, ab108319, Abcam), anti-Lin28A antibody (1:500, 16177-1-AP, Proteintech Group Inc.), anti-Lin28B antibody (1:500, 16178-1-AP, Proteintech Group Inc.), anti-Dicer antibody (1:500, NB200-591, Novus Biologicals LLC, Littleton, CO, USA), anti-Bcl-2 antibody (1:1000, 12789-1-AP, Proteintech Group Inc.), anti-Bax antibody (1:1000, 50599-2-Ig, Proteintech Group Inc.), anti-active caspase-3 (1:500, ab2302, Abcam), and anti-β-actin antibody (1:1000, 20536-1-AP, Proteintech Group Inc.). Thereafter, the membranes were washed with PBS and incubated with peroxidase-conjugated goat anti-rabbit IgG (1:2000; SA00001-2, Proteintech Group Inc.) at 25° C for 1 h. The membranes were then visualized using ECL reagent (P0018, Beyotime Co.). The immunoblots were analyzed using ImageJ software (version 1.46r; National Institutes of Health, Bethesda, MD, USA). Eight mice from each group were collected for western blot analysis.

### Optomotor response

Optomotor response was evaluated as previously described [50, 51]. Seven-day RIR injured mice were placed on a platform in the form of a grid (20.0 cm diameter, 25.0 cm above the bottom of the drum) surrounded by a motorized drum (20.0 cm diameter) that could be revolved clockwise or counterclockwise at a constant speed of 12 degrees per second, the optimal velocity for evoking an optokinetic response in the mouse. After 5 min of adaptation to a light of 400 lux, vertical black and white stripes (100% contrast) of a defined spatial frequency were presented to the animal. The protocol yielded independent measures of right and left eye acuity based on the unequal sensitivity of the two eyes to pattern rotation: the right and left eyes are most sensitive to counterclockwise and clockwise rotations, respectively. The test was initiated by presenting the mouse with a sinusoidal striped pattern that rotated either clockwise or counterclockwise and had varied widths. These stripes were rotated clockwise and counterclockwise, alternately, for 2 min in each direction with an interval of 30 s between rotations. The spatial frequencies tested were 0.05, 0.1, 0.2, 0.3, 0.4, 0.5, and 0.6 cycles per degree (cpd). Animal performance was recorded with a digital video camera for subsequent scoring of head tracking movements. Eight mice from each group were collected for the optomotor response test.

### Light-dark exploration testing

This test was performed as described previously [52], with adaptations. A seven-day RIR injured mouse was initially placed in the dark chamber and its activity was quantified for 5 min by determining the time spent in the illuminated chamber. Eight mice from each group were collected for the light-dark exploration test.

### Statistical analysis

Data are expressed as mean ± standard error of the mean (SEM). Statistical analysis was performed by Student’s *t*-test or one-way analysis of variance followed by Tukey’s test where appropriate. GraphPad Prism software (version 6.01; GraphPad Software Inc., San Diego, CA, USA) was used for the analysis. Statistical significance was considered at *p* < 0.05.
